# Biomarkers Correlate With Body Composition and Performance Changes Throughout the Season in Women's Division I Collegiate Soccer Players

**DOI:** 10.3389/fspor.2020.00074

**Published:** 2020-07-02

**Authors:** Bridget A. McFadden, Alan J. Walker, Michelle A. Arent, Brittany N. Bozzini, David J. Sanders, Harry P. Cintineo, Marissa L. Bello, Shawn M. Arent

**Affiliations:** ^1^Department of Exercise Science, The University of South Carolina, Columbia, SC, United States; ^2^IFNH Center for Health and Human Performance, Rutgers University, New Brunswick, NJ, United States; ^3^Department of Exercise Science, Lebanon Valley College, Annville, PA, United States; ^4^Department of Health Promotion, Education, and Behavior, The University of South Carolina, Columbia, SC, United States

**Keywords:** catabolic, anabolic, athlete-monitoring, female athlete, performance testing

## Abstract

The purpose of this study was to evaluate the effects of a competitive soccer season on biomarkers and performance metrics in order to determine the correlation between changes in biomarkers, body composition, and performance outcomes. Twenty-one Division 1 female collegiate soccer players were monitored throughout the 16-week season. Player workload was measured using heart rate and Global Position Satellite systems at all practices and games. Performance testing, including vertical jump, VO_2max_, and 3-repetition maximum testing for bench press, squat and deadlift, occurred prior to pre-season and immediately post-season. Blood draws occurred prior to preseason and every 4-weeks thereafter, following a game. Body composition was assessed prior to the start of season (week 0) and weeks 6, 10, 14, and 17 (post-season). Delta area under the curve was calculated for biomarkers and body composition variables to account for seasonal changes adjusted for baseline. Pearson-product moment correlations were used to assess relationships with significance set at *p* < 0.05. Trends were considered *p* ≤ 0.10. No significant time main effects were seen for anabolic biomarkers (*p* > 0.05). Significant time effects were seen for catabolic biomarkers throughout the season (*p* = 0.001). No changes in body weight, VO_2max_, vertical jump, and deadlift occurred. Squat and bench press improved (*p* = 0.01 and *p* = 0.02, respectively) with a decline in percent body fat (*p* = 0.03) and a trend for increased fat free mass (*p* = 0.09). Additionally, total cortisol (TCORT) negatively correlated with fat free mass (*r* = −0.48; *p* = 0.03) and positively correlated with VO_2max_ (*r* = 0.47; *p* = 0.04). A trend was shown for a positive correlation between both TCORT and free cortisol (FCORT) and percent body fat (*r* = 0.39; *r* = 0.40; *p* = 0.08, respectively). IGF-1 and growth hormone positively correlated to deadlift (*r* = 0.57; *P* = 0.02 and *r* = 0.59; *p* = 0.03), whereas creatine kinase showed a trend for a positive correlation with deadlift (*r* = 0.49; *p* = 0.06). IL-6 negatively correlated with bench press (*r* = −0.53; *p* = 0.03). These findings support a relationship between biomarkers, performance outcomes, and body composition. Biomarker monitoring may be useful to detect individual player's physiological response to an athletic season and may help provide insights in efforts to optimize performance outcomes.

## Introduction

The physiological demands of soccer require athletes to possess high levels of aerobic capacity, as well as muscular strength, muscular power, speed, and speed-endurance (Kraemer et al., 2004; Stolen et al., 2005). Collegiate soccer players face unique challenges to maintaining these metrics throughout the competitive season. Specifically, multiple games per week, frequent practices, and travel requirements can increase these athletes' overall stress and recovery needs (Walker et al., 2019b). Moreover, changes in diet, environment, and sleep combined with the burdens of academic requirements that often accompany collegiate life can increase athletes' risk for injury and further hinder the ability to enhance aspects of sport performance that are required to succeed (Silva, 1990; Meeusen et al., 2013; Mann et al., 2015). Therefore, it is important to employ athlete monitoring techniques that assess the cumulative impact of these stressors, not simply the physical stress due to the sport, in order to better facilitate adequate recovery and optimal performance. Monitoring techniques that track changes in an athlete's physiological state may prove beneficial in determining an athlete's readiness to perform (Huggins et al., 2019; Walker et al., 2019b).

The implementation of systematic performance assessments is one important method of athlete monitoring that plays a key role in the determination of athlete readiness. Systematic assessments, more specifically, periodic maximal performance tests, are essential in determining an individual athlete's capabilities, as well as monitoring the effectiveness of training (Krustrup et al., 2005; Stolen et al., 2005; Sporis et al., 2009). For power-endurance athletes, these tests may include maximal oxygen consumption (VO_2max)_ tests that measures the highest flow of oxygen that can be used by the tissues in order to determine aerobic capacity (Taylor et al., 1955), maximal vertical jump (VJ) height to assess muscular power, and repetition maximum (RM) tests for muscular strength (Walker et al., 2019a). While maximal performance tests are optimal to determine player development, the potential for added fatigue incurred by the athletes as well as the time burden required to evaluate an entire team, makes these tests difficult to implement during the season in a team sport environment (Bangsbo and Lindquist, 1992; Twist and Highton, 2013; Halson, 2014; Thorpe et al., 2015). In a condensed collegiate season marked by high training volumes, adding an additional load of performance assessments periodically throughout the season may not be feasible, as coaches and support staff would be required to concede already limited sport specific training/practice times to implement testing. Therefore, it is important to incorporate other assessment methods that do not incur additional physical loads on the athletes, can be implemented without burdening coaches, and allow for continuous monitoring throughout the season (Thorpe et al., 2015). The use of workload tracking systems, such as heart rate (HR) and Global Navigation Satellite System (GNSS) metrics (or global positioning satellite (GPS) systems specific to the United States), throughout the season plays an important role in assessing recovery needs, detecting non-functional overreaching, reducing risk of injury, and ultimately maximizing athletic performance (Halson, 2014). While these workload tracking systems can detect an athlete's response to on-field training stressors, the use of biomarker monitoring offers the ability to track an individual player's physiological response to *overall* stress as well as to identify the balance between training and recovery (Lee et al., 2017; Pedlar et al., 2019).

Periodic biomarker assessments during the competitive soccer season offer an accurate, objective method for evaluating training related stress and recovery needs as well as overall athlete health (Bessa et al., 2016; Pedlar et al., 2019). The use of biomarkers may better indicate exercise related stress independent of other factors, such as motivation, which can confound the results of performance tests or other subjective measures (Bessa et al., 2016). Although there is no single definitive biomarker that can be used to monitor training progress or recovery status in team sport athletes (Marques-Jimenez et al., 2016; Lee et al., 2017), measuring biomarkers that are indicative of an athlete's physiological state may prove to be a useful tool in determining athlete readiness. Kraemer et al. (2004), proposed that if the physical demands of training are too high or there is insufficient recovery to meet the training demands, a catabolic state will persist throughout the season, resulting in impaired performance. Markers associated with catabolism, such as total and free cortisol (TCORT and FCORT), creatine kinase (CK), c-reactive protein (CRP), and interleukin-6 (IL-6), are often released in response to excessive strain or muscle damage (Lee et al., 2017). Elevations in these catabolic markers have been shown to promote inflammation and muscle breakdown and chronically high values may indicate impaired recovery (Lee et al., 2017) thus affecting performance outcomes (Kraemer et al., 2004). However, if training demands are met with adequate recovery, an anabolic environment will result in the maintenance or improvement of performance (Kraemer et al., 2004). For example, the anabolic hormone testosterone, promotes an anticatabolic effect and may improve athletic performance through direct effects on muscle growth, enhanced muscle repair after exercise, accelerated neuromuscular coordination, and the regulation of body fat (Koundourakis and Margioris, 2019). Other anabolic hormones relevant to the female athlete such as, insulin-like growth factor-1 (IGF-1) and growth hormone (GH), have been shown to promote muscle growth and development of muscle tissue (Frystyk, 2010; Nindl and Pierce, 2010; Lee et al., 2017) and may provide additional insights in regards to muscular adaptations to training. Estradiol (E_2_) is a form of the hormone estrogen, which is responsible for female sex characteristics and has been shown to have various beneficial effects on athletic performance through anti-inflammatory properties and improved muscle recovery and repair rates (Tiidus, 2003; Koundourakis and Margioris, 2019). Estrogen may exert a protective effect on muscle tissue during exercise by acting as an antioxidant, membrane stabilizer, and through receptor substrate characteristics (Cano Sokoloff et al., 2016; Koundourakis and Margioris, 2019). Additionally, alterations in E_2_ have been shown to be reflective of an energy deficiency and suggest hypothalamic-pituitary-gonadal (HPG) axis dysfunction in the female athlete (Dueck et al., 1996; Vanheest et al., 2014). Overall, chronic elevations in anabolic hormones may lead to improvements in body composition and performance, whereas declines in these markers may reflect impaired potential for adaptations to training (Lee et al., 2017).

Changes in hormonal concentrations in conjunction with changes in exercise performance during the course of a soccer training program have been previously reported in male athletes (Filaire et al., 2003; Kraemer et al., 2004; Silva et al., 2008; Huggins et al., 2019). Over the course of a competitive season, Kraemer et al. (2004) demonstrated decrements in sprint speed, vertical jump height, and knee extensor strength. These downturns in performance coincided with elevated concentrations of circulating cortisol and reduced testosterone. Together these findings indicate a predominance of catabolic processes occurred during the season (Kraemer et al., 2004). In another study in professional soccer players, testosterone was found to have a direct relationship between vertical jump height and power output suggesting its beneficial effects on athletic performance, whereas a negative correlation was seen between endurance capacity and both serum cortisol and testosterone levels (Bosco et al., 1996; Koundourakis and Margioris, 2019). Silva et al. (2008) demonstrated increases in average team CK levels coincided with diminished team performance (win percentage) as well as stagnation in aerobic endurance (Silva et al., 2008). Similarly, Filaire et al. (2003) examined changes in various biomarkers throughout a season and found that decreases in glutamine and increases in cortisol overlapped with a decreased win percentage; however, immunological and hematological markers, VO_2max_ and body composition remained unchanged (Filaire et al., 2003). In a comprehensive study by Huggins et al. (2019), 92 separate biomarkers were analyzed at five time points throughout a collegiate men's competitive soccer season. Although fluctuations in biomarkers were observed, these fluctuations were interpreted to be typical changes that occur over the course of a season within a team setting (Huggins et al., 2019). Furthermore, the team experienced little change in player load between training blocks which coincided with slight decreases in GPS and accelerometry metrics as well as no significant changes in body composition, VO_2max_, or HR at anaerobic threshold. In one of the only studies evaluating biomarker changes in female soccer athletes, researchers demonstrated significant declines in VO_2max_, vertical jump, body weight and body fat percentage from pre- to post-season which occurred along with increases in various catabolic biomarkers including cortisol, IL-6, and CK (Walker et al., 2019b). The decrements in performance metrics in conjunction with sustained increases in catabolic biomarkers from preseason may be a result of the accumulated stress of the season thereby negatively impacting performance variables.

Although it is apparent that changes in biomarkers may occur during a female soccer season along with performance decrements, it is unclear if changes in specific anabolic and catabolic markers correlate to changes in performance outcomes. In addition, identifying and utilizing athlete-monitoring methods, such as biomarkers, that are associated with performance outcomes may allow for prompt player evaluation and may also be useful in designing individualized recovery strategies (de Hoya et al., 2016; Pedlar et al., 2019; Walker et al., 2019b). Therefore, the purpose of this study was to determine the impact of a National Collegiate Athletic Association (NCAA) Division I (DI) women's soccer season on anabolic and catabolic biomarkers in conjunction with various measures of fitness and performance. Additionally, this study sought to identify the relationship between biomarkers, performance, and body composition changes in female collegiate soccer athletes over the course of a competitive season. It was hypothesized that as a team, the athletes would experience increased catabolic markers and declines in anabolic markers throughout the season, and decrements in performance metrics would occur from pre- to post-season. However, when assessing individual changes in concentrations of biomarkers from baseline, greater change in catabolic markers would negatively correlate with performance outcomes and fat free mass (FFM), while changes in anabolic markers would be positively associated with performance and FFM.

## Materials and Methods

### Participants

Women's DI college soccer players (*N* = 21; Age = 20 ± 2 y; Weight = 66.3 ± 6.2 kg, Height = 169 ± 7 cm) participated in monitoring and assessments throughout the course of a competitive soccer season. The team consisted of six defenders, six forwards, seven midfielders, and two goal keepers. The 16-week season, which began in early August and finished in mid-November, corresponded with the end of summer period and lasted until mid-fall in the Northeast region of the United States. Athletes performed training and performance testing as part of regular team activities associated with their sports science program. During this time, athletes participated in a periodized strength and conditioning (S&C) program one time per week set by the team's strength coach in addition to on-field agility training sessions incorporated into practice. The strength training program focused on power and strength development with sessions consisting of 1–2 power movements followed by 3–5 strength movements and associated core work. Power movements consisted of exercises such as the high pull and push press. Strength movements consisted of exercises including back squat, split squat, bench press, incline dumbbell press, Romanian deadlifts, rack pulls, single arm dumbbell rows, and associated variations of these movements. Weekly loads were programmed based on preseason 3 RM strength testing results and consisted of 70–80% of their estimated 1RM (Brzycki, 1993). All subjects received clearance by the University's Sports Medicine staff prior to testing and at the start of the season. This research was approved, and written consent was waived by the Rutgers University Institutional Review Board for the Protection of Human Subjects (IRB#16-050M). All procedures performed were in accordance with the 1964 Declaration of Helsinki and its later amendments or comparable ethical standard.

### Experimental Design and Procedures

**Figure 1:**
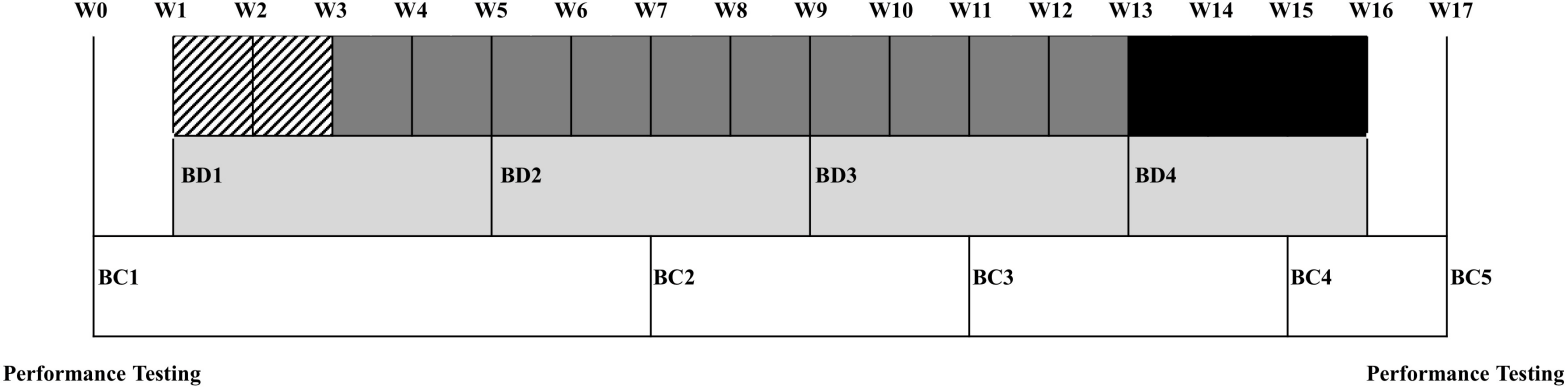
Timeline of assessments. BC, Body Composition Testing; BD, Blood Draws. Strip boxes represents 2-weeks of preseason, dark gray boxes represent weeks of the regular season, black boxes represent tournament time, light gray boxes represent time between blood draws, white boxes represent time between body composition testing.

This study sought to evaluate the effects of a competitive collegiate soccer season on biomarkers and performance metrics in order to determine the correlation between biomarkers, body composition, and performance outcomes in DI female soccer players. Biomarkers were analyzed prior to the start of preseason and every 4-weeks following the initial analysis. Maximal performance testing occurred prior to the start of the season and within 1-week following the final match. Body composition testing occurred prior to the start of preseason, at weeks 6, 10, 14, and within 1-week following the final match. See Figure 1 for a timeline of assessments.

### Performance Testing

Athletes reported to the IFNH Center for Health and Human Performance (CHHP) prior to the start of preseason (W0) (late July) and again within 1-week following the final competitive match (W17) (mid-November) to complete a battery of fitness tests to assess body composition, VO_2max_, and muscular strength during a 1-week period. Subjects were instructed to arrive euhydrated, 2–3 h fasted, having abstained from caffeine, and having performed no exercise 24 h prior to testing. Body composition was assessed by air displacement plethysmography (Dempster and Aitkens, 1995) (BOD POD, COSMED, Concord, CA, USA) to determine percent body fat (%BF), and fat free mass (FFM) using the Brozek formula (Brozek et al., 1963). In addition to W0 and W17, body composition was also assessed at weeks 6, 10, and 14. Next, following a 10–15 min generalized dynamic warm up, subjects performed maximal countermovement vertical jumps with both hands-on-hips (VJ_HOH_) and with arm swing (VJ) that was assessed using the Just Jump Mat (Probotics, Huntsville, AL, USA). Subject were given three attempts for each jump variation, with the highest jump for each recorded. The error of vertical jump height for females has been reported with a standard error of the mean of 1.7 cm and a CV of 4.4% (Nuzzo et al., 2011). After completing power testing, a treadmill-based maximal graded exercise test (GXT) was used to measure maximal aerobic capacity (VO_2max_) via direct gas exchange using an indirect calorimeter (Quark CPET, COSMED, Concord, CA, USA). A speed-based protocol was used with stages that were MET-equated to the Bruce protocol. This protocol included 2 min stages at a constant 2% incline. The speeds were as follows: 6.4, 7.9, 10.0, 11.7, 13.7, 15.6, 17.1, 18.2, 19.8, 21.1 (km/h) (McFadden et al., 2020). Subjects continued the test with encouragement from lab staff until volitional fatigue. At least three of the following criteria were met verifying attainment of VO_2max_: a leveling off or plateauing of VO_2_ with an increase in workload, attainment of age predicted heart rate max, a respiratory exchange ratio >1.10, and/or an RPE ≥18 (Thompson et al., 2000). Heart rate was continuously monitored using a Polar S610 heart rate monitor (Polar Electro Co., Woodbury, NY, USA) to obtain maximal heart rate (HR_max_). One player (*n* = 1) did not participate in post season VO_2*max*_ testing due to clearance from sport medicine.

On a separate day, maximal strength testing was performed within a group setting under the instruction and supervision of the team's strength and conditioning staff. Following a generalized dynamic warmup, 3RM tests were completed for squat (SQ), bench press (BP), and deadlift (DL) in that respective order and in accordance with NSCA guidelines (Haff and Triplett, 2016). Four players did not participate in post-season strength testing due to professional team opportunities (*n* = 1), job requirements (*n* = 1), or clearance from sports medicine (*n* = 2).

### Biomarker Collection and Analysis

Athletes were instructed to report to the CHHP in a euhydrated condition immediately prior to the start of pre-season for blood draws (BD1), and every 4-weeks following (BD2, BD3, and BD4). All blood draws were taken at the CHHP between 0700 and 0830 h following an overnight fast, and BD2, BD3, and BD4 draws occurred ~18–36 h following a game (Walker et al., 2019b). Blood samples were obtained (antecubital fossa, 21G, BD Vacutainer, Safety-Lok) from the antecubital vein in the arm by an experienced phlebotomist while subjects were seated. Whole blood was collected and left to clot at room temperature. Samples were drawn into collection tubes consisting of anticoagulant (EDTA), or clot activator (gel free and Serum Separator Tubes). Following collection, blood was centrifuged for 10 min at 4,750 rpm (Allegra x-15R, Beckman Coulter, Brea, CA, USA). Plasma and serum samples were shipped (in containers designed to maintain approximately 20, 4, or −20°C depending on the analyte) to a processing facility (Quest Diagnostics, Inc., San Juan Capistrano, CA, USA) for analysis. Samples were run in duplicate and the coefficient of variation (CV) was between 0.5 and 10.0% for all biomarkers. Biomarkers analyzed included CK, CRP, TCORT, FCORT, E_2_, GH, IGF-1, IL-6, free testosterone (FTEST), and total testosterone (TTEST).

### Training Load

**Figure 2:**
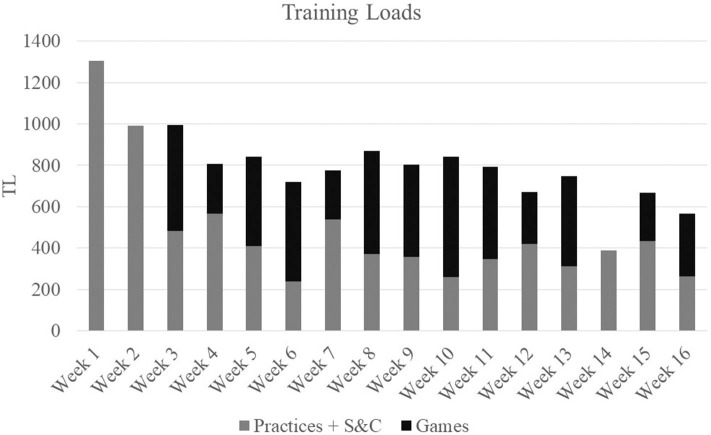
Total weekly training loads.

**Figure 3:**
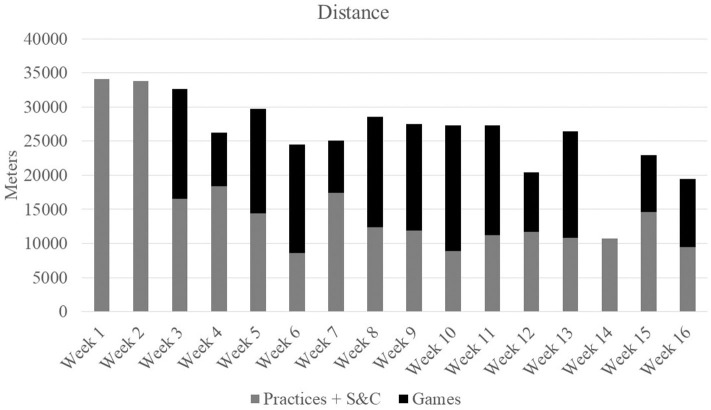
Total weekly distances.

**Figure 4:**
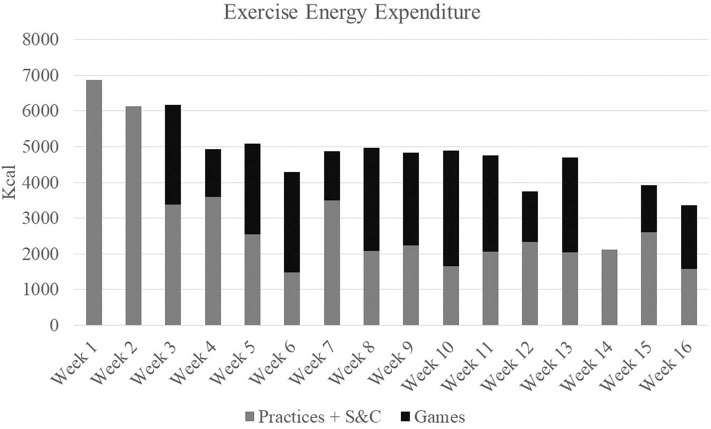
Total weekly exercise energy expenditures.

Athlete workloads were monitored at all practices, games, and strength sessions throughout the entirety of the 16-week season which consisted of 21 games, 63 practice sessions, and 15 strength sessions. Workload was monitored through HR and GPS tracking technology using the Polar TeamPro system (Polar Electro Co., Woodbury, NY, USA). Physiological attributes of the player obtained from laboratory testing (age, height, body weight, sex, VO_2max_, HR_max_, and ventilatory threshold) were used to program each individual player's monitor. Training load (TL), exercise energy expenditure (EEE), and distance covered (DIS) were used to quantify the team's total weekly workload (Ceesay et al., 1989). TL was calculated via an algorithm developed by Polar^TM^ based on physiological attributes of the player, obtained from laboratory testing, and physical workload measures comprised of HR, GPS, and accelerometry information (Ceesay et al., 1989; Giersch et al., 2018). Weekly team workloads are presented in Figures 2–4.

### Statistical Analysis

Pre- and post-season performance and body composition data were analyzed using repeated measures (RM) multivariate analysis of variances (MANOVAS) with RM analysis of variance univariate follow-ups using a listwise deletion factor. RM MANOVAS were used to assess biomarker changes throughout the season. For each univariate analysis, the Huynh-Feldt epsilon was examined for the general model to evaluate sphericity. If the Huynh-Feldt epsilon exceeded 0.75, sphericity was considered to have been met, and the unadjusted statistic was used. If epsilon was <0.75, the adjusted Huynh-Feldt statistic was used to test significance. Planned simple contrasts were conducted using the baseline values as the comparison term. Pairwise contrasts were included in the case of significant univariate findings using the least significant difference method. Delta area under the curve (DAUC) was calculated for the biomarkers and body composition variables using the trapezoidal method to account for seasonal changes adjusted for baseline. Pearson-product moment correlations (r) were used to assess the relationships between biomarker changes and changes in performance and body composition. All analyses were conducted using SPSS (version 23, IBM, Armonk, NY, USA) with significance set at *p* < 0.05. Trends were considered to be *p* ≤ 0.10. Shapiro-Wilks test was used to test normality. In the case of non-normality of the data, variables were log transformed for analysis. An r value of 0.36–0.67 were considered to represent a moderate correlation. An r value of 0.68–1.0 were considered to represent a strong correlation (Weber and Lamb, 1970). Cohen's *d* was used to calculate effect sizes (ES) from baseline (P1, BC1, and BD1). Using Cohen's conventions, ES of 0.20, 0.50, and 0.80 were considered indicative of small, medium, and large effects, respectively. Biomarker and performance variables are presented and expressed as means ± standard deviation. Percent change (Δ) is represented by means and standard error. All individual raw biomarker and performance data is provided in the Supplemental Material.

## Results

As a team, no changes in VO_2max_, VJ, VJ_HOH_, or DL performance were seen from W0 to W17 (*p* > 0.05). However, significant improvements in BP (ΔBP = 1.6 ± 0.7 kg; *p* = 0.02) and SQ (ΔSQ = 3.55 ± 1.08 kg; *p* = 0.01) performance were seen. Performance changes pre- to post-season are expressed in Table 1. No change in body weight was seen from W0 to W17 (*p* > 0.05) (Table 2). A significant decline in %BF was seen from W0 to W17 (Δ%BF = −1.4 ± 0.6%; *p* = 0.03) with a trend for an increase in FFM (ΔFFM = 0.59 ± 0.3 kg; *p* = 0.09) (Table 2).

**Table 1:** Preseason to postseason performance changes.

**Performance**	**Preseason**	**Postseason**	**ES**
VO_2Max_ (mL·kg·min^−1^)	47.5 ± 4.0	47.8 ± 3.9	0.08
Vertical Jump_HOH_ (cm)	45.3 ± 5.3	46.4 ± 5.6	0.21
Vertical Jump (cm)	50.2 ± 6.7	50.6 ± 6.4	0.06
3RM Bench Press (kg)	43.2 ± 7.2	44.8 ± 5.1^*^	0.22
3RM Squat (kg)	80.9 ± 12.8	84.5 ± 12.2^*^	0.28
3RM Deadlift (kg)	85.3 ± 15.2	84.4 ± 11.3	−0.06

*Values are expressed as means and standard deviations*.

* *Indicates a significant difference from pre to post*.

**Table 2:** Preseason to postseason body composition changes.

**Body Composition**	**Preseason**	**Postseason**	**ES**
FFM (kg)	53.76 ± 6.2	54.35 ± 5.9^#^	0.10
% BF	18.88 ± 4.7	17.44 ± 5.2^*^	−0.31
Weight (kg)	66.26 ± 6.2	65.84 ± 6.0	−0.07

*Values are expressed as means and standard deviations*.

* *Indicates a significant difference from pre to post*.

# *Indicates a trend for a difference from pre to post*.

RM MANOVAS showed no significant time main effects with anabolic biomarkers (GH, IGF-1, TTEST, FTEST, E_2_) over the course of the season (*p* > 0.05). However, significant time main effects were found for the catabolic biomarkers (TCORT, FCORT, CK, IL-6, CRP) (*p* = 0.001). Univariate follow-ups revealed significant changes in FCORT throughout the season (*p* = 0.01) (Changes from BD1 are presented in Table 3).

**Table 3:** Biomarker Changes.

**Biomarkers**	**BD1 ± SD**	**BD2 ± SD**	**ES_**1−2**_**	**BD3 ± SD**	**ES_**1−3**_**	**BD4 ± SD**	**ES_**1−4**_**
IGF-1 (μg/L)	320.65 ± 88.69	303.45 ± 90.8	−0.19	293.80 ± 74.31	−0.30	299.65 ± 74.84	−0.24
GH (μg/L)	2.97 ± 3.61	3.34 ± 2.52	0.10	1.99 ± 3.12	-0.27	2.92 ± 3.69	−0.01
TTEST (nmol/L)	0.96 ± 0.05	0.94 ± 0.04	−0.02	0.88 ± 0.05	-1.66	0.96 ± 0.04	0.03
FTEST (pmol/L)	7.51 ± 4.89	8.14 ± 4.21	0.13	7.44 ± 4.71	-0.01	7.91 ± 3.95	0.82
E_2_ (pmol/L)	271.95 ± 246	146.62 ± 101.43^#^	−0.51	235.80 ± 223.68	-0.15	307.91 ± 388.22	0.15
TCORT (nmol/L)	626.85 ± 288.93	672.09 ± 317.32	0.16	655.40 ± 219.87	0.10	646.71 ± 285.91	0.07
FCORT nmol/L	34.86 ± 12.54	26.43 ± 7.36^*^	−0.67	34.02 ± 10.80	-0.07	40.88 ± 12.10	0.48
CK (U/L)	368.00 ± 297.24	379.35± 348.51	0.04	364.70 ± 815.50^*^	-0.01	170.80 ± 106.14^*^	−0.66
IL-6 (pg/mL)	0.80 ± 0.27	1.10 ± 0.65^#^	1.04	1.71 ± 1.88^*^	3.33	1.27 ± 1.82	1.72
CRP (mg/L)	0.76 ± 0.81	2.16 ± 3.51^*^	1.73	2.66 ± 5.74^#^	2.35	0.92 ± 0.98	0.20

*Values are expressed as means and standard deviations*.

* *Indicates a significant difference from baseline*.

# *Indicates a trend for a difference from baseline*.

Biomarkers that correlate with performance and body composition variables are presented in Table 4. TCORT was negatively correlated with FFM (*r* = −0.48, *p* = 0.03). TCORT and FCORT also showed a positive correlation with %BF that approached significance (*r* = 0.39; *r* = 0.40; *p* = 0.08, respectively). TCORT was positively correlated with VO_2max_ (*r* = 0.47; *p* = 0.04) while a trend for a positive association with FCORT and VO_2max_ was seen (*r* = 0.39; *p* = 0.09). IL-6 was negatively correlated with BP (*r* = −0.53; *p* = 0.03) while IGF-1 and GH positively correlated with DL (*r* = 0.57; *p* = 0.03 and *r* = 0.59; *p* = 0.02, respectively). A trend for a positive correlation between CK and DL was seen (*r* = 0.49; *p* = 0.06). A positive correlation between TTEST and DL approached significance (*r* = 0.50; *p* = 0.07). No significant correlations were seen for biomarkers and VJ/VJ_HOH_ (*p* > 0.05). No significant correlations were seen between CRP, FTEST, and measures of body composition or performance (*p* > 0.05).

**Table 4:** Biomarkers and performance/body composition correlations.

		**FFM**	**%BF**	**BW**	**SQ**	**BP**	**DL**	**VO_**2max**_**	**VJ**	**VJ_**HOH**_**
TCORT	*r*	−0.477^*^	0.390^#^	0.089	−0.4	0.004	0.245	0.467^*^	0.26	0.032
FCORT	*r*	−0.2	0.40^#^	0.29	0.183	0.036	0.06	0.39^#^	0.059	0.023
CK	*r*	0.021	−0.187	−0.198	−0.013	−0.391	0.491^#^	0.011	0.025	0.19
E_2_	*r*	0.198	−0.276	−0.205	0.181	−0.128	0.421	−0.165	−0.304	−0.277
GH	*r*	−0.002	−0.085	−0.092	0.195	0.333	0.593^*^	0.211	−0.131	−0.256
CRP	*r*	0.135	−0.076	0.027	0.364	−0.33	−0.281	0.148	0.287	0.347
IGF-1	*r*	−0.027	−0.05	−0.114	0.338	−0.028	0.567^*^	0.119	−0.182	−0.278
IL-6	*r*	0.112	−0.088	−0.014	0.298	−0.534^*^	−0.18	0.043	0.077	0.303
FTEST	*r*	0.309	0.014	0.251	0.242	0.124	0.318	−0.086	−0.179	−0.291
TTEST	*r*	−0.005	0.088	0.068	0.091	−0.064	0.497^#^	0.272	−0.078	−0.23

* *significant correlation between biomarkers and performance*.

# *trend toward significant correlation between biomarkers and performance*.

Within the biomarker analysis, TCORT positively correlated with FCORT (*r* = 0.63; *p* = 0.002). A negative correlation between TCORT and E_2_ trended toward significance (*r* = −0.36; *p* = 0.10). GH was positively correlated with IGF-1 (*r* = 0.48; *p* = 0.03). IGF-1 positively correlated with FTEST (*r* = 0.60; *p* < 0.001) and E_2_ (*r* = 0.49; *p* = 0.02). IL-6 positively correlated with CRP (*r* = 0.76; *p* < 0.001). The positive correlation between E_2_ and FTEST approached significance (*r* = 0.45, *p* = 0.06). Correlations between biomarkers are presented in Table 5.

**Table 5:** Biomarker correlations.

		**TCORT**	**FCORT**	**CK**	**E_**2**_**	**GH**	**CRP**	**IGF-1**	**IL-6**	**FTEST**	**TTEST**
TCORT	*r*	1									
FCORT	*r*	0.63^*^	1								
CK	*r*	0.148	−0.068	1							
E_2_	*r*	−0.362^#^	−0.326	0.133	1						
GH	*r*	0.301	0.306	−0.317	0.02	1					
CRP	*r*	0.05	0.187	0.082	−0.044	−0.127	1				
IGF-1	*r*	0.045	0.184	−0.002	0.491^*^	0.480^*^	−0.122	1			
IL-6	*r*	0.108	0.314	0.099	0.041	−0.177	0.756^*^	−0.03	1		
FTEST	*r*	−0.13	0.097	−0.197	0.426^#^	0.287	−0.055	0.600^*^	0.113	1	
TTEST	*r*	0.22	0.154	0.097	0.253	0.325	0.003	0.718^*^	0.082	0.747^*^	1

* *significant correlation between performance markers*.

# *trend toward significant correlation between performance markers*.

Within performance and body composition measures, a positive correlation was seen between body weight and BP (*r* = 0.56; *p* = 0.02). Trends were seen for a negative correlation between VO_2max_ and FFM (*r* = −0.37; *p* = 0.10) and a positive correlation with %BF (*r* = 0.42; *p* = 0.065). VJ_HOH_ positively correlated with VJ (*r* = 0.56; *p* = 0.01). Performance and body composition correlations are presented in Table 6.

**Table 6:** Performance and body composition correlations.

		**FFM**	**%BF**	**BW**	**SQ**	**BP**	**DL**	**VO_**2max**_**	**VJ**	**VJ_**HOH**_**
FFM	*r*	1								
%BF	*r*	−0.533^*^	1							
BW	*r*	0.205	0.711^*^	1						
SQ	*r*	0.024	−0.192	0.172	1					
BP	*r*	0.269	0.253	0.563^*^	−0.075	1				
DL	*r*	0.039	−0.323	−0.318	−0.042	−0.058	1			
VO_2max_	*r*	−0.373^#^	0.420^#^	0.062	−0.156	−0.124	−0.091	1		
VJ	*r*	−0.329	0.227	0.057	0.362	0.167	−0.188	0.095	1	
VJ HOH	*r*	−0.265	0.035	−0.157	0.291	−0.329	−0.343	0.203	0.560^*^	1

* *significant correlation between biomarkers*.

# *trend toward significant correlation between biomarkers*.

## Discussion

As a team, over the course of a 16-week soccer season, which was punctuated by high TL, EEE, and DIS, female collegiate soccer athletes experienced alterations in catabolic markers and a maintenance of anabolic markers. Additionally, improvements in body composition, increases in strength, and a preservation of both aerobic capacity and power metrics from pre- to post-season occurred in these athletes. After adjusting for individual baseline values, positive associations were seen between anabolic biomarkers (GH and IGF-1) and DL strength. Additionally, greater overall increases in markers of stress and inflammation (IL-6 and cortisol), were negatively associated with BP strength and FFM measures. Interestingly, net increases in TCORT from baseline were also positively correlated with VO_2max_. These findings support a relationship between cumulative changes in anabolic and catabolic markers and changes in performance and body composition in female collegiate athletes throughout a competitive season, and therefore can be useful in the evaluation of athlete adaptation to training and readiness to perform.

In the current study, fluctuations were seen in catabolic markers at various timepoints indicating varying levels of stress occurred in these athletes throughout the season. The highest CK values occurred at the first two timepoints and declined as the season progressed. Though within typical female athlete reference ranges (Mougios, 2007), elevated CK levels at BD2 coincided with the highest TLs, EEE, and DIS of the season. Further, elevations in both IL-6 and CRP were apparent at BD2 which persisted before returning to baseline values at the end of the season. A lack of adequate recovery from soccer-related training stressors may result in a pro-inflammatory state exhibited by high concentrations of cytokines, such as IL-6 and CRP. IL-6 in particular, has been shown to increase in response to decreased muscle glycogen and muscle contraction (Pedersen and Febbraio, 2005), and is often associated with muscle damage (Bessa et al., 2016), diminished muscular strength, and physical function (Ferrucci et al., 2002; Stenholm et al., 2010). This may be evident in the negative correlation between IL-6 and BP in the current study. In addition to cytokines and CK, cortisol has been shown to be reflective of a catabolic state (Lee et al., 2017). In the current study, fluctuations in FCORT were seen throughout the season. Although no changes in TCORT values were noted, values at each time point were considered above the normal clinical reference ranges (127–569 nmol/L when assessed 0800–1,000 h) which indicates a catabolic state was evident in these athletes prior to the start of the season and remained as the season progressed. Chronic elevations in cortisol may make it difficult for an athlete to build or maintain muscle or recover from training (Lee et al., 2017), a relationship that was reflected in the negative correlations with TCORT and FFM and the moderate positive associations between TCORT, FCORT and %BF.

Although greater net elevations in TCORT showed a negative correlation with FFM, interestingly, net elevations in TCORT were also positively correlated with VO_2max_. This may be indicative of a power/endurance tradeoff (Kraemer et al., 1995) typically noted in times of increased aerobic activity that might be seen during high volumes of soccer-specific training. Intensive aerobic training can improve endurance performance reflected by improvements in VO_2max_ (Bresciani et al., 2011). However, if intensive aerobic training is not met with adequate recovery, altered hormonal responses including increases in cortisol may persist. The positive correlation between VO_2max_ and TCORT may be a result of an overload of aerobic training, although more research is needed to determine the underlying mechanism behind this observed relationship. It is also interesting to note that a trend was seen for a moderate positive association between CK and DL. This relationship may be reflective of the work completed by the athletes, as increases in CK may be indicative of muscle breakdown and remodeling which could result in improvements in strength if coupled with a proper anabolic stimulus.

As a team, anabolic markers remained relatively stable despite the fluctuations in catabolic markers. The maintenance of anabolic markers over the course of the season, coincided with the preservation of both power and endurance as well increases in strength. Greater concentrations of anabolic hormones may help to promote protein synthesis and counteract muscle damage that may occur during soccer (Hackney and Machado, 2012). Whereas, low levels of key anabolic hormones, such as GH and IGF-1, that play a role in regulating muscle growth (Frystyk, 2010) have been associated with low muscle mass and strength (Araujo et al., 2008; Stenholm et al., 2010). This is potentially evidenced by greater individual changes in GH and IGF-1 measures positively correlated with DL in this study. In addition, a moderate positive correlation occurred between DL and TTEST.

For the female athlete, one area of particular interest is E_2_ and its relationship to performance outcomes. Although no correlations between E_2_ and performance or body composition were seen, overall elevations in TCORT showed a moderate negative correlation with E_2_. Lowered E_2_ in conjunction with increased TCORT has been associated with energy deficiencies caused by an inadequate caloric intake relative to exercise energy expenditures (Mountjoy et al., 2018). Low energy availability is shown to alter levels of various hormones leading to disruptions in optimal health and performance (Vanheest et al., 2014; Mountjoy et al., 2018). Further, E_2_ was positively correlated with IGF-1, while correlations between E_2_ and FTEST approached significance. IGF-1 has been shown to reflect energy status, with a combination of low energy intake and high volumes of training shown to reduce IGF-1 (Zanker and Swaine, 2000). Ultimately, the positive correlation between increased IGF-1 and E_2_ may be the result of a more favorable anabolic environment (Nindl and Pierce, 2010; Koundourakis and Margioris, 2019), while declines in IGF-1 in conjunction with E_2_ may be a response to an energy deficit (Nemet et al., 2004; Vanheest et al., 2014; Joro et al., 2017) associated with the high caloric expenditures characteristic of soccer athletes. More research is warranted to determine the impact of energy availability on biomarker changes as well as performance outcomes in order to identify individuals in need of a nutrition intervention. It is worth noting that although biomarker monitoring did occur every 4-weeks to account for a typical menstrual cycle, the individual menstrual status of the athletes was not “controlled.” Given the nature of this applied study, researchers sought to determine a typical hormonal environment that exists for a collegiate team during the season. Additionally, since menstrual cycle does not control playing time, we believe that this is an accurate representative profile of a female collegiate soccer team.

In addition to lack of reported information on athlete's menstrual status, the authors acknowledge several other limitations to this current study which may have impacted biomarker results. First, diet was not controlled throughout the season. Researchers sought to determine typical biomarker responses that occur in these athletes without nutritional controls. Although the findings would have benefited from the addition of dietary information, accuracy and adherence challenges associated with dietary logs limit the feasibility of this method in a team setting. In addition, sleep and mood states have also been shown to be associated with physiological responses and performance outcomes (Morgan et al., 1987; Filaire et al., 2001; Halson, 2014). Evaluating subjective measures of sleep and mood may provide useful context to interpreting physiological responses to training. However, it is important to consider the burden of implementing additional testing procedures to collegiate athletes who already experience heightened responsibilities inherent with collegiate student athlete life. Future research may consider ways to address these concerns without overburdening the athlete. Further, although consistent with an average soccer team's roster, the small sample size in the current study may limit the interpretation of these results. Future research may be warranted to determine biomarker and performance associations in female soccer athletes with similar training requirements across multiply teams.

Finally, it is important to consider that increases and decreases in a single biomarker often do not occur independently as illustrated by various convergent hormonal pathways (Lee et al., 2017). This is further evident in the correlations amongst biomarkers in this study, such as the associations of CRP and IL-6, IGF-1, and GH, along with testosterone and IGF-1. Therefore, rather than looking at one biomarker to determine the effects of training load on an athlete, it may be more advantageous to analyze multiple anabolic and catabolic biomarkers together when assessing adaptations to a training program.

The results of this study support a relationship between biomarkers, body composition, and performance outcomes in female collegiate soccer athletes. Despite the accumulated stressors, as a team, performance metrics were maintained or modestly improved in the case of SQ and BP performance. In addition, individual elevations in anabolic hormones from baseline were related to improvements in measures of strength, specifically DL performance, whereas greater net elevations in catabolic or inflammatory markers were related to declines in FFM and BP. Further, TCORT was positively correlated with VO_2max_ and negatively correlated with FFM indicating that an overload of aerobic soccer-specific training may have predominated in certain players during the season. It appears that biomarker monitoring may be a useful tool in team sports to determine an individual athlete's recovery needs and player readiness status. Monitoring changes in anabolic and catabolic markers throughout a season may provide an opportunity to intervene before decrements in performance occur. Changes in an athlete's physiological state can provide context for coaches and support staff to manipulate training variables in order to promote recovery or provide a greater stimulus to enhance adaptations needed to optimize performance. Additionally, biomarker monitoring may be useful to detect individual's physiological response to a given training load and serve as a guide to strength and conditioning coaches regarding periodization of strength training programs to optimize performance. Improved knowledge of the athlete's physiological state may allow for more informed decisions regarding the application of training loads by coaches and trainers to promote athlete performance and adaptation to training. This may include modifying volume and intensity of training, programmed rest and recovery, improved nutritional strategies, and substitution strategies that promote better player management (Marques-Jimenez et al., 2016). Additionally, the use of multiple biomarkers may better detect training stressors related to recovery status and performance outcomes rather than relying on performance tests alone. Through properly structured strength and conditioning programs in conjunction with appropriate assessment methods and application of training loads, it may be possible to avoid the power/endurance tradeoff and loss of FFM often seen over the course of the season in these athletes.

## Data Availability Statement

All datasets generated for this study are included in the article/Supplementary Material.

## Ethics Statement

The studies involving human participants were reviewed and approved by Rutgers University Institutional Review Board for the Protection of Human Subjects. Written informed consent for participation was not required for this study in accordance with the national legislation and the institutional requirements.

## Author Contributions

BM, AW, and SA conceived and designed the experiments. BM, AW, DS, BB, HC, MB, MA, and SA performed the experiments. BM, AW, BB, and SA analyzed the data. SA contributed materials/tools. BM, BB, MA, HC, and SA wrote the paper.

## Conflict of Interest

The authors declare that the research was conducted in the absence of any commercial or financial relationships that could be construed as a potential conflict of interest.
